# Automated Grading of Vesicoureteral Reflux (VUR) Using a Dual-Stream CNN Model with Deep Supervision

**DOI:** 10.1007/s10278-025-01438-1

**Published:** 2025-02-14

**Authors:** Guangjie Chen, Lixian Su, Shuxin Wang, Xiaoqing Liu, Wenqian Wu, Fandong Zhang, Yijun Zhao, Linfeng Zhu, Hongbo Zhang, Xiaohao Wang, Gang Yu

**Affiliations:** 1 Department of Urology, National Clinical Research Center for Child Health, The Children’s Hospital, Zhejiang University School of Medicine, Hangzhou, China; 2 Deepwise Artificial Intelligence Laboratory, Beijing, China; 3 National Clinical Research Center for Child Health, The Children’s Hospital, Zhejiang University School of Medicine, Hangzhou, China

**Keywords:** Vesicoureteral reflux, Voiding cystourethrography, Multi-head convolutional neural network, Dual-stream architecture

## Abstract

Vesicoureteral reflux (VUR) is a urinary system disorder characterized by the abnormal flow of urine from the bladder back into the ureters and kidneys, often leading to renal complications, particularly in children. Accurate grading of VUR, typically determined through voiding cystourethrography (VCUG), is crucial for effective clinical management and treatment planning. This study proposes a novel multi-head convolutional neural network for the automatic grading of VUR from VCUG images. The model employs a dual-stream architecture with a modified ResNet-50 backbone, enabling independent analysis of the left and right urinary tracts. Our approach categorizes VUR into three distinct classes: no reflux, mild to moderate reflux, and severe reflux. The incorporation of deep supervision within the network enhances feature learning and improves the model’s ability to detect subtle variations in VUR patterns. Experimental results indicate that the proposed method effectively grades VUR, achieving an average area under the receiver operating characteristic curve of 0.82 and a patient-level accuracy of 0.84. This provides a reliable tool to support clinical decision-making in pediatric cases.

## Introduction

Vesicoureteral reflux (VUR) is a urinary system disorder where urine abnormally flows back from the bladder into the ureters and even the kidneys [1]. Under normal conditions, urine flows from the kidneys to the bladder through the ureters. VUR is commonly diagnosed in infants and children and is frequently associated with renal complications such as renal scarring, hypertension, and chronic end-stage renal disease [2]. Therefore, the timely diagnosis of VUR is critical for preserving kidney health in children.

Accurate grading of VUR is essential for effective treatment planning and clinical management. Internationally, VUR is graded on a scale from 0 to 5, where grade 0 indicates no reflux and grade 5 represents the most severe form. Children with lower-grade VUR (grades 1–3) typically do not require surgical intervention and are often monitored for spontaneous resolution. In contrast, children with higher-grade VUR (grades 4–5) usually require prompt surgical intervention and anti-inflammatory medications to reduce the risk of urinary tract infections [3, 4].

Voiding cystourethrography (VCUG) is the primary imaging tool used for the clinical diagnosis and grading of VUR. VCUG imaging relies on the use of iodinated contrast agents, such as iothalamate meglumine [5], and typically covers the entire urination cycle to detect the presence of reflux [6]. In addition to anteroposterior imaging, lateral imaging may be performed to further evaluate ureter morphology.

Despite the clear definitions for VUR grading based on VCUG images [7], the process remains challenging, particularly for less experienced clinicians, due to significant variations in ureter morphology across patients and the potential for subjective judgment. Some studies have focused on developing quantitative parameters for VUR grading from VCUG images, such as the diameter of the distal ureter [8] and delayed post-voiding contrast drainage of the upper urinary tract [9]. Additionally, artificial intelligence (AI) techniques, including pattern recognition, have been employed to automate VUR grading and minimize subjectivity. Eroglu et al. [10] proposed a hybrid approach for diagnosing and grading VUR from VCUG images, integrating convolutional neural networks for feature extraction, minimum redundancy maximum relevance for feature selection, and support vector machines (SVMs) for classification. While promising, this method is limited by its reliance on SVMs, which struggle to capture complex, non-linear relationships between features, a significant drawback given the high variability of medical images. Li et al. [11] introduced the Deep-VCUG model, which comprises a lateral classification module based on ResNet-101 and a VUR grade classification module leveraging an ensemble of five weak models. A voting mechanism is then applied to generate the final classification result. While ensemble methods can enhance performance by mitigating bias and variance, the use of weak models, each with inherently low predictive power, may not provide sufficient improvement in classification accuracy.

In this study, we propose a novel multi-head convolutional neural network for automatic VUR grading from VCUG images. The key innovation of this model is the use of multiple classification heads, each responsible for independently analyzing the left and right urinary tracts, combined with deep supervision to improve feature learning and classification accuracy.

## Materials and Methods

### Dataset Characteristics

We retrospectively collected 1529 cases of pediatric patients, each associated with one VCUG image. The dataset was divided into two subsets: 1229 images for training and 300 images for testing. The detailed label distribution is presented in Table 1. The ground truth for VUR grading was established based on the International Reflux Society classification criteria [12], with two expert pediatric urologists determining the accurate labels for the dataset.

**Table 1:** Dataset distribution

VUR categories	VUR grading	Right ureter		Left ureter	
		Training set (*N* = 1229)	Test set (*N* = 300)	Training set (*N* = 1229)	Test set (*N* = 300)
Class 0: no reflux	Grade 0	526 (42.8%)	101 (33.7%)	545 (44.3%)	94 (31.3%)
Class 1: mild to moderate reflux	Grade 1	83 (6.8%)	12 (4.0%)	88 (7.2%)	16 (5.3%)
	Grade 2	98 (8.0%)	37 (12.3%)	122 (9.9%)	31 (10.3%)
	Grade 3	129 (10.5%)	13 (4.3%)	127 (10.3%)	32 (10.7%)
Class 2: severe reflux	Grade 4	165 (13.4%)	57 (19.0%)	145 (11.8%)	63 (21.0%)
	Grade 5	228 (18.6%)	80 (26.7%)	202 (16.4%)	64 (21.3%)

### Data Processing

All VCUG images were initially cropped to focus on the bladder and ureteral regions, resulting in a final image resolution of 768 × 768 pixels. To enhance image contrast, histogram equalization was applied individually to the red, green, and blue channels of each image. To increase the diversity of the training set and enhance model robustness, data augmentation was employed through random flipping with 50% of probability. This straightforward yet effective method improved the model’s generalization by introducing variations in image orientation.

### Methods

Our approach consisted of a multi-stage process to comprehensively assess VUR and its clinical implications. The first stage involved a binary classification task, distinguishing between the absence of VUR (grade 0) and the presence of VUR. This step provided an initial indication of whether the patient exhibited VUR.

In the second stage, a more refined three-way classification was performed. This classification grouped VUR grades into three categories: no reflux (grade 0), mild to moderate reflux (grades 1–3), and severe reflux (grades 4–5), where the latter often indicates the need for surgical intervention. This refined classification aimed to better guide clinical decision-making by identifying patients who may benefit from surgical treatment.

Both classification tasks were addressed using our proposed multi-head model, which independently analyzed the left and right urinary tracts. This multi-head architecture enhanced the model’s ability to capture subtle differences in VUR patterns and ensured accurate grading.

### Model Details

**Fig. 1:**
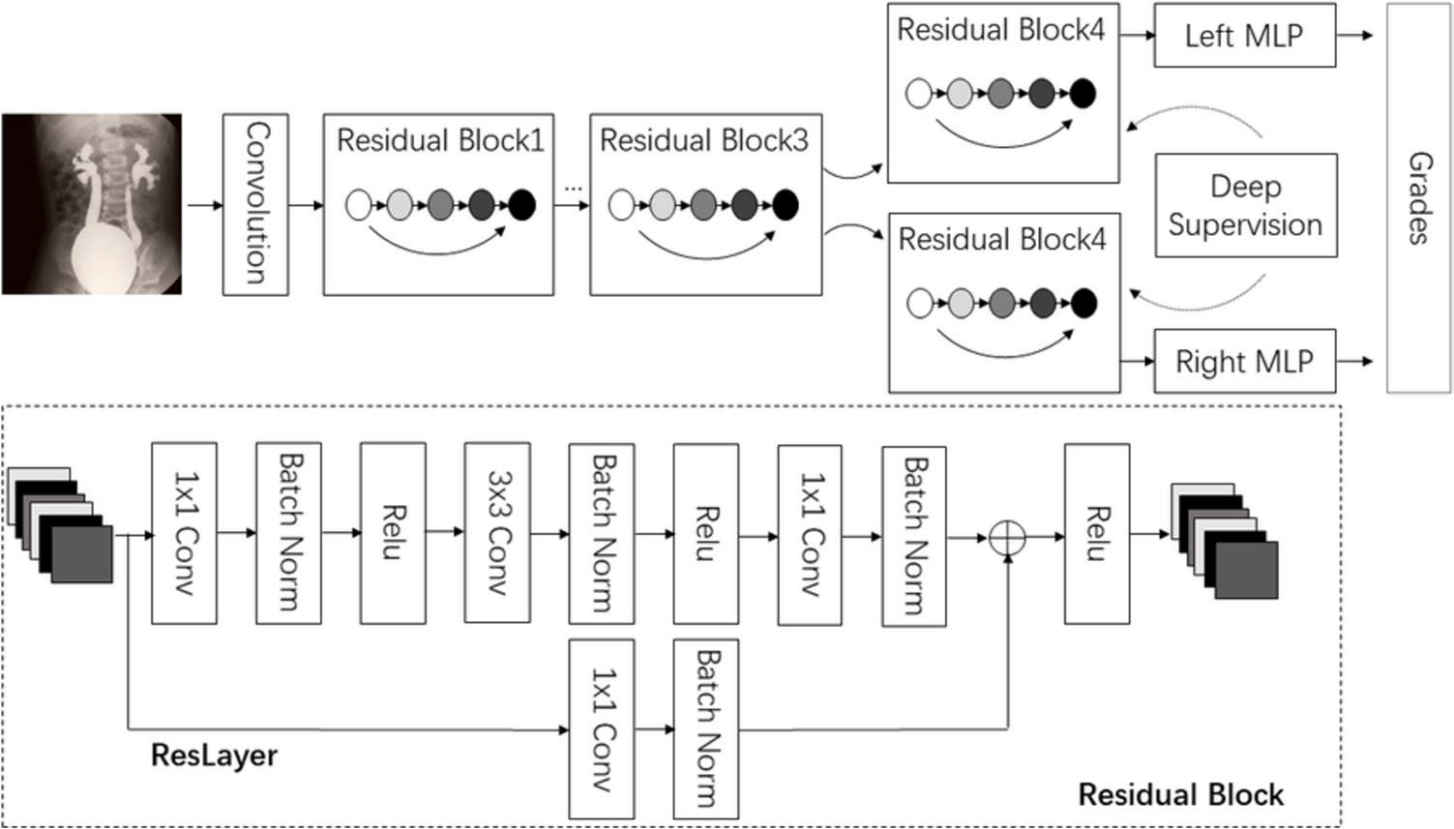
Structure of proposed model

As shown in Fig. 1, the proposed model features multi-head processing of the left and right bladder images using a dual-stream architecture built on a modified ResNet-50 backbone [13], combined with deep supervision to improve feature learning.

The base of our model consists of the first three blocks of a pre-trained ResNet-50 [13], which processes the input images and extracts feature maps through several convolutional and pooling layers. To separately process the left and right bladder images, we duplicated the fourth block of ResNet-50, creating two parallel processing streams. One stream is dedicated to extracting features from the left bladder images, while the other focuses on the right bladder images, allowing the model to learn distinct features from each side.

To further enhance feature learning, the model incorporates a deep supervision mechanism. Before processing the final block, feature maps are extracted from the third block of ResNet-50 [13]. These intermediate features are flattened and passed through a fully connected layer, which provides auxiliary outputs. The deep supervision mechanism enables the model to learn more discriminative features early in the process, potentially improving final classification accuracy.

After the parallel processing streams, we apply an average pooling layer to reduce spatial dimensions and aggregate the feature maps. The pooled features from each stream are then flattened and passed through multi-layer perceptron (MLP). We utilize two MLPs for each stream to map the high-dimensional features to the desired number of output labels.

The loss function used in the model combines the primary classification loss with the deep supervision loss [14]. The primary classification loss is computed using a focal loss [15]}, applied to the predicted grades for the left and right bladder images to address class imbalance and emphasize harder-to-classify examples during training, as shown by the following equation:

1 $${L}_{CLS}={\sum }_{\text{c}}{y}_{\text{true},\text{c}}{\alpha }_{\text{c}}{\left(1-{p}_{\text{pred},\text{c}}\right)}^{\gamma }\text{log}\left({p}_{\text{pred},\text{c}}\right)$$

where *y*_truc,*c*_ represents the true label for class c, *p*_pred,*c*_ is the predicted probability for class c, *α*_c_ is a balancing factor for the positive or negative class c, and *γ* is a tunable focusing parameter that controls the down-weighting of well-classified examples.

To incorporate the deep supervision mechanism, we compute the cross-entropy loss on the intermediate features extracted from the third block of ResNet-50. These intermediate losses, derived from the deep supervision fully connected layer, provide additional guidance to the model during training, as shown in the following equation:

2 $${L}_{DS}=-{\sum }_{\text{c}}{y}_{\text{true},\text{c}}\text{log}\left({p}_{\text{pred},\text{c}}\right)$$

where *y*_true,c_ represent the true label for class c and *p*_pred,c_ is the predicted probability for class c.

The total loss is the weighted sum of the primary classification loss and the deep supervision loss, ensuring a balanced contribution from both components. The total loss function is defined as follows:

3 $${L}_{\text{total}}=\alpha {L}_{CLS}+\left(1-\alpha \right){L}_{DS}$$

where *α* is a weighting factor that balances the contributions of the classification loss and the deep supervision loss.

### Implementation Details

Training was conducted using the AdamW optimizer with an initial learning rate of 0.00002 and a weight decay of 0.01 over 24 epochs. The batch size was set to 16 per device, and mixed-precision training (fp16) was employed to enhance computational efficiency. The data loader utilized four worker threads to expedite data loading. Checkpoints were saved at the end of each epoch, retaining only the most recent one to conserve storage space.

All code was implemented in Python and PyTorch. The experiments were performed on a workstation equipped with four NVIDIA TITAN RTX GPUs (24 GB GPU memory each), 256 GB of RAM, and an Intel Xeon Gold 6248 CPU at 2.50 GHz, running Ubuntu 16.04.

## Results

### Model Performance Evaluation

**Fig. 2:**
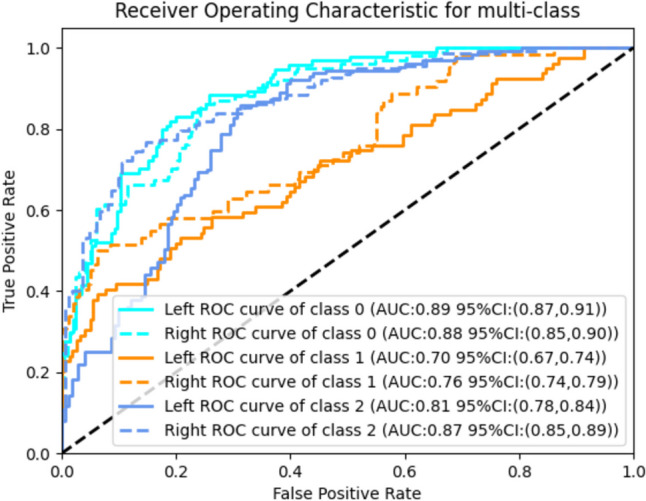
ROC curve for multi-class

We evaluated the performance of the proposed model using several metrics, including accuracy, sensitivity, specificity, and the area under the receiver operating characteristic (ROC) curve (AUC). The multi-class ROC curve, presented in Fig. 2, illustrates the model’s ability to discriminate among the three categories: Class 0 (no reflux), Class 1 (mild to moderate reflux), and Class 2 (severe reflux). The model achieved an average AUC of 0.82, indicating strong overall performance in classification tasks.

Table 2 presents the performance metrics for each category on both the right and left ureters, along with *p*-values assessing differences between the two sides. No significant differences are observed in the classification of “no reflux” and “mild to moderate” cases, indicating that the model performs consistently across both ureters. However, for “severe reflux,” significant differences are noted in both accuracy and specificity, with the right ureter demonstrating slightly better performance. This suggests that the model’s effectiveness may vary depending on the side, particularly in severe cases. A possible explanation could be class imbalance or data distribution bias, which may favor the right ureter, leading to more accurate classifications on that side.

**Table 2:** Experimental results

Categories	Right ureter			Left ureter			*P*-value between right-left ureter		
	Acc. (95%CI)	Sen. (95%CI)	Spec. (95%CI)	Acc. (95%CI)	Sen. (95%CI)	Spe. (95%CI)	Acc	Sen	Spe
Class 0: no reflux	0.80 (0.77–0.86)	0.86 (0.79–0.93)	0.79 (0.74–0.85)	0.80 (0.76–0.85)	0.86 (0.80–0.93)	0.79 (0.74–0.84)	0.493	0.910	0.658
Class 1: mild to moderate	0.86 (0.84–0.91)	0.52 (0.39–0.65)	0.97 (0.94–0.99)	0.86 (0.84–0.90)	0.52 (0.38–0.64)	0.97 (0.94–0.98)	0.332	0.472	0.724
Class 2: severe reflux	0.80 (0.77–0.85)	0.77 (0.71–0.84)	0.84 (0.78–0.89)	0.73 (0.70–0.80)	0.72 (0.65–0.80)	0.77 (0.70–0.83)	0.033	0.179	0.014
Weighted Avg	0.82 (0.80–0.86)	0.75 (0.70–0.80)	0.85 (0.82–0.88)	0.80 (0.78–0.82)	0.70 (0.66–0.74)	0.81 (0.78–0.85)	0.224	0.437	0.635

**Fig. 3:**
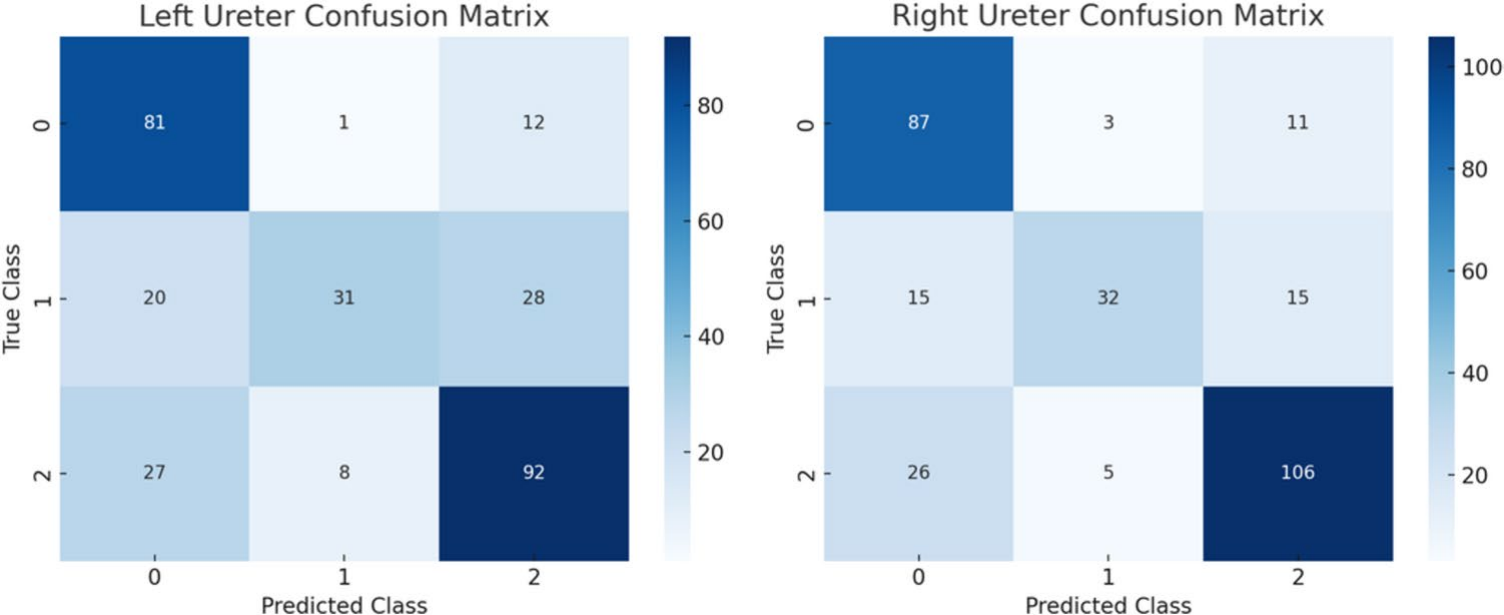
Confusion matrices

Additionally, the confusion matrices depicted in Fig. 3 provide a detailed breakdown of the model’s predictions versus the actual labels. The heatmaps highlight areas where the model performs well and where mis-classifications occur. Notably, the model shows high accuracy in identifying cases of no reflux and severe reflux but exhibits reduced sensitivity in detecting mild to moderate reflux. This reduced sensitivity is primarily attributed to the limited amount of training data available for this category compared to others. Statistical analysis of the dataset revealed that grades 1–3, corresponding to mild to moderate reflux, had the fewest examples, which likely hindered the model’s ability to learn effective patterns for this grade. These results suggest that while the model is reliable for detecting no reflux and severe reflux cases, improvements are needed to enhance sensitivity for mild to moderate reflux detection. Future work may involve collecting more data, especially for grades 1–3, and/or incorporating data augmentation strategies to address class imbalance.

### Ablation Study

In our method, focal loss was employed to address class imbalance and to focus on harder-to-classify examples, significantly improving the model’s performance in challenging cases. Additionally, the multi-head classification approach allowed the model to independently classify images of the left and right ureters, facilitating the learning of distinct features for each side. Deep supervision was incorporated to guide the model’s learning using auxiliary outputs from intermediate feature maps, further enhancing feature representation.

To validate the effectiveness of each component, we conducted ablation experiments. These experiments tested variations of the model by removing deep supervision, modifying the multi-head classification, and using different loss functions. The results are summarized in Table 3.

**Table 3:** Ablation experiments

Experiments	Right ureter (avg)			Left ureter (avg)			Patient level
	Acc./*P*-value (95%CI)	Sen./*P*-value (95%CI)	Spe./*P*-value (95%CI)	Acc./*P*-value (95%CI)	Sen./*P*-value (95%CI)	Spe./*P*-value (95%CI)	Acc./*P*-value
No Deep Sup	0.75/0.041 (0.71–0.77)	0.69/0.049 (0.65–0.75)	0.83/0.028 (0.80–0.86)	0.71/0.037 (0.67–0.75)	0.63/0.059 (0.61–0.67)	0.80/0.028 (0.77–0.82)	0.77/0.002
No Multi-Head	0.81/0.034 (0.77–0.84)	0.70/0.043 (0.67–0.77)	0.85/0.024 (0.81–0.89)	0.78/0.032 (0.74–0.81)	0.65/0.053 (0.59–0.70)	0.80/0.024 (0.78–0.84)	0.79/0.009
No Focal Loss	0.76/0.031 (0.72–0.81)	0.68/0.040 (0.66–0.72)	0.84/0.022 (0.79–0.88)	0.72/0.030 (0.69–0.77)	0.62/0.048 (0.58–0.66)	0.81/0.023 (0.77–0.84)	0.80/0.032
Ours	0.83/–- (0.81–0.86)	0.72/–- (0.67–0.77)	0.87/–- (0.84–0.89)	0.79/–- (0.75–0.82)	0.66/–- (0.61–0.70)	0.83/–- (0.81–0.86)	0.84/–-

The ablation studies demonstrated that removing or altering any of these components led to a noticeable decline in model performance. Specifically, the removal of deep supervision resulted in a significant reduction in the model’s ability to learn discriminative features at early stages, highlighting the importance of this mechanism in improving overall classification accuracy. Similarly, the multi-head classification approach, which allowed for independent processing of the left and right urinary tracts, proved essential for capturing subtle anatomical differences. Removing this feature impaired the model’s ability to accurately classify VUR grades. Furthermore, focal loss was found to be crucial for addressing the inherent class imbalance in the dataset. By focusing attention on harder-to-classify cases, focal loss significantly improved the robustness and reliability of the model’s predictions.

Additionally, the patient-level accuracy shows statistically significant improvements with the inclusion of deep supervision and multi-head classification, as indicated by the *p*-values *p* < 0.05 in Table 3. Here, patient-level accuracy is calculated based on the most severe reflux category present on either side of the ureters.

Overall, these ablation studies underscore the importance of each network component in the proposed model. The combination of deep supervision, multi-head classification, and focal loss was critical to achieving optimal performance, with each component playing a distinct and complementary role in the architecture.

### Comparison with State-of-the-Art Methods

Li et al. [11] developed a Deep-VCUG model for VUR classification, incorporating MobileNetv2, GoogLeNet, ResNet-101, DenseNet161, and EfficientNet-B0. To ensure a direct comparison, we trained and tested these five models using our dataset, with the results presented in Table 4. Our model consistently outperformed these approaches at both the ureter and patient levels, achieving higher overall accuracy and a more balanced trade-off between sensitivity and specificity. Furthermore, the *p*-values for our model’s accuracy at the patient level indicate statistical significance, further reinforcing its robustness. These findings suggest that our model offers superior accuracy and generalizability for VUR classification compared to existing methods.

**Table 4:** Comparisons with state-of-the-art methods

Experiments	Right ureter (avg)			Left ureter (avg)			Patient level
	Acc./*P*-value (95%CI)	Sen./*P*-value (95%CI)	Spe./*P*-value (95%CI)	Acc./*P*-value (95%CI)	Sen./*P*-value (95%CI)	Spe./*P*-value (95%CI)	Acc./*P*-value
MobilNetv2	0.78/0.015 (0.73–0.82)	0.69/0.073 (0.61–0.78)	0.83/0.069 (0.78–0.87)	0.77/0.005 (0.73–0.80)	0.63/0.037 (0.53–0.66)	0.79/0.052 (0.74–0.84)	0.82/0.032
GoogLeNet	0.74/0.038 (0.69–0.79)	0.64/0.017 (0.62–0.68)	0.81/0.274 (0.74–0.89)	0.74/0.027 (0.70–0.79)	0.62/0.182 (0.56–0.69)	0.78/0.018 (0.74–0.86)	0.78/0.043
ResNet101	0.74/0.263 (0.67–0.81)	0.68/0.140 (0.64–0.75)	0.84/0.020 (0.81–0.88)	0.76/0.029 (0.72–0.80)	0.64/0.032 (0.59–0.68)	0.78/0.014 (0.74–0.83)	0.79/0.024
DenseNet161	0.75/0.058 (0.73–0.78)	0.65/0.084 (0.59–0.71)	0.84/0.094 (0.79–0.89)	0.77/0.276 (0.72–0.79)	0.64/0.013 (0.59–0.68)	0.80/0.104 (0.76–0.82)	0.80/0.027
EfficientNet-B0	0.71/0.007 (0.66–0.76)	0.62/0.019 (0.55–0.67)	0.80/0.019 (0.73–0.84)	0.72/0.047 (0.68–0.77)	0.60/0.026 (0.56–0.67)	0.76/0.002 (0.72–0.83)	0.75/0.008
Ours	0.83/–- (0.81–0.86)	0.72/–- (0.67–0.77)	0.87/–- (0.84–0.89)	0.79/–- (0.75–0.82)	0.66/–- (0.61–0.70)	0.83/–- (0.81–0.86)	0.84/–-

### Attention Map

**Fig. 4:**
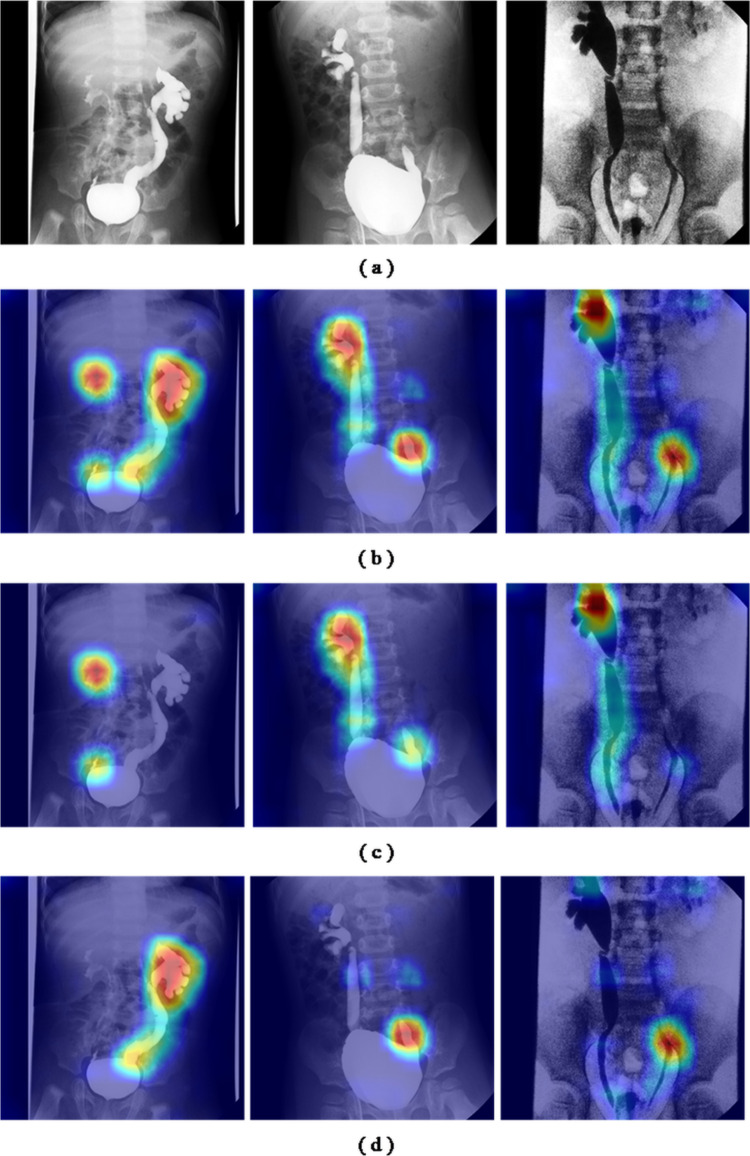
A visualization of the proposed model is presented. The top row displays the original VCUG images, while the bottom row shows the corresponding heatmaps, where red indicates regions of higher activation and blue indicates regions of lower activation, highlighting the ureteral areas of interest. **a** Original VCUG images. **b** The model’s complete attention maps. **c** Attention maps for the left ureter branch. **d** Attention maps for the right ureter branch

To gain deeper insights into whether the model was focusing on relevant anatomical regions, we utilized gradient-weighted class activation mapping (Grad-CAM) [16] to visualize the internal features of the neural network. The findings revealed that the neural network predominantly focused on the ureteral region when grading VUR. The visualization results are shown in Fig. 4.

The heatmap visualizations indicate that the model’s dual-stream architecture effectively enables the left and right branches to concentrate on their respective sides of the image, ensuring that each branch processes the correct anatomical features without interference.

Additionally, the model demonstrated its ability to extract relevant features from both frontal and posterior views of the bladder, underscoring its robustness in handling different perspectives. Importantly, the Grad-CAM results suggest that the model can accurately identify ureteral regions and, in some instances, detect subtle instances of reflux that may be challenging for human clinicians to observe. These findings imply that the model not only understands the spatial relationships critical for VUR grading but also enhances the potential for early detection of clinically significant features.

## Discussion

In this study, we present a multi-head convolutional neural network for automatic VUR grading from VCUG images. Compared to existing methods, our model offers greater computational efficiency, enhanced ability to capture complex data relationships, and the advantage of deep supervision for more consistent and robust feature learning. We reduce redundancy by integrating left and right ureter analyses within a unified architecture, enabling the model to learn shared features across both sides. This design improves classification accuracy while maintaining computational efficiency.

One key limitation of this study is the relatively small dataset, which constrains the level of granularity in the classification system that could be implemented. We adopted a three-class classification system for VUR (grades 0, 1–3, and 4–5) rather than a more granular six-class system. Implementing a six-class classification would have required a larger and more diverse dataset to ensure robust and reliable model performance across all categories. On the other hand, the chosen three-class system still provides meaningful insights, particularly in differentiating between cases that require surgical intervention (grades 4–5) and those that do not (grades 0 and 1–3). This approach enables the model to focus on clinically significant thresholds while addressing challenges related to class imbalance and data limitations. By consolidating VUR grades into three broader categories, we aimed to balance maintaining clinically relevant distinctions with ensuring reliable model performance.

Expanding the dataset will be a priority in future work. A larger dataset would allow us to explore more granular classification schemes, including the possibility of developing a full six-class system. With a more extensive and diverse dataset, the model could be trained to recognize finer distinctions between VUR grades, further enhancing its clinical utility.

Additionally, future efforts will focus on integrating advanced data augmentation techniques and transfer learning strategies to improve the model’s generalization capabilities, even with limited data. Another potential avenue for exploration is the incorporation of multi-modal data. For instance, combining imaging data with patient demographics and clinical history could lead to the development of a more comprehensive model capable of providing personalized treatment recommendations.

## Conclusion

This study presents a dual-stream model designed to classify vesicoureteral reflux (VUR) by first detecting its presence and then assessing its severity into three categories. Incorporating deep supervision significantly enhances feature learning, while the use of focal loss effectively addresses class imbalance by focusing on more challenging cases.

Ablation experiments confirm the importance of each model component, particularly the multi-head classification architecture and deep supervision. The model demonstrated robust performance across key metrics, including high accuracy, sensitivity, specificity, and AUC values, along with strong patient-level accuracy. This approach provides a valuable tool for clinical decision-making, especially in identifying patients who may require surgical intervention.
